# Cholinergic stimulation stabilizes TRPM4 in the plasma membrane of cortical pyramidal neurons

**DOI:** 10.3389/fcell.2024.1440140

**Published:** 2024-07-23

**Authors:** Paula Leyton, Denise Riquelme, Francisco A. Peralta, Franco D. Navarro, Elias Leiva-Salcedo

**Affiliations:** Department of Biology, Faculty of Chemistry and Biology, University of Santiago, Santiago, Chile

**Keywords:** TRPM4, traffic, cortical neuron, FRAP, cholinergic

## Abstract

TRPM4 is a calcium activated non-selective cation channel, impermeable to Ca^2+^, in neurons it has been implicated in the regulation of the excitability and in the persistent firing. Cholinergic stimulation is also implicated in changes in excitability that leads neurons to an increased firing frequency, however it is not clear whether TRPM4 is involved in the cholinergic-induced increase in firing frequency. Here using a combination of patch clamp electrophysiology, Ca^2+^ imaging, immunofluorescence, fluorescence recovery after photobleaching (FRAP) and pharmacological approach, we demonstrate that carbachol (Cch) increases firing frequency, intracellular Ca^2+^ and that TRPM4 inhibition using 9-Ph and CBA reduces firing frequency and decreases the peak in intracellular Ca^2+^ induced by Cch in cortical pyramidal neurons in culture. Moreover, we determined that cholinergic stimulation reduces TRPM4 recycling and stabilizes TRPM4 in the plasma membrane. Together our results indicate that cholinergic stimulation increases firing in a TRPM4 dependent manner, and also increases the TRPM4 stability in the membrane, suggesting that TRPM4 is locked in microdomains in the membrane, possibly signaling or cytoskeleton proteins complexes.

## Introduction

Neuronal intrinsic excitability is established by the number and distribution of ion channels, it determines action potential (AP) firing and synaptic transmission properties, and it coordinates neuronal activity, thus controlling the information flow through the circuits. The intrinsic excitability is a dynamic process finely regulated by neurotransmitters and neuromodulators such as the cholinergic transmission, which modulates neuronal excitability through the inhibition of the M currents driven by KCNQ2/3 channels (Bordas et al., 2015; Lezmy et al., 2017; Ye et al., 2022), by the afterdepolarization, through the activation of TRPC and TRPM (Haj-Dahmane and Andrade, 1999; McQuiston and Madison, 1999; Smith and Araneda, 2010; Dasari et al., 2013; Kim et al., 2013; Lei et al., 2014; Carver et al., 2021) or by increasing the activity of the G protein-coupled inwardly rectifying potassium channels (GIRK) (Luo et al., 2022), among other mechanisms. Overall, ion channels and neurotransmitters act together to modulate information flow.

Cholinergic stimulation activates an afterdepolarization potential dependent on calcium-activated non-selective cation currents (I_CAN_) that triggers persistent firing in pyramidal neurons of the prefrontal cortex layer 5 (Haj-Dahmane and Andrade, 1999). Similarly, Lei et al. (2014) determined that the Transient Receptor Potential Melastatin 4 (TRPM4), an I_CAN_ channel, has a small contribution in the carbachol induced afterdepolarization (ADP) in the layer five pyramidal neurons of the prefrontal cortex. Moreover, Kim et al. (2013) reported a depolarization-induced slow current dependent on TRPM4 which participates in the depolarization after potential in cerebellar Purkinje neurons. Similarly, in layer 2/3 pyramidal neurons, TRPM4 is activated after a train of depolarization and induces an afterdepolarization which increases the AP firing (Riquelme et al., 2021). Recently, Combe et al. (2023), determined that TRPM4 modulates the AP adaptation dependent on muscarinic activation in CA1 pyramidal neurons, shifting from earlier to later firing, suggesting that cholinergic modulation could trigger place cell firing. Overall, TRPM4 enhances excitability and increases AP firing in various neuronal populations, implicating its involvement in neural circuit dynamics and potentially cognitive processes like spatial representation.

Changes in channel expression and trafficking are critical for determining the excitable properties of neurons and their response to synaptic stimuli. In this regard, Kuba et al. (2010) determined that auditory deprivation increases Nav current and the axon initial segment (AIS) length. Similarly, it also reduces Kv1 and increases Kv7 in the AIS (Kuba et al., 2015), thereby reducing shunting conductance and increasing neuronal excitability. Similarly, glutamate stimulation induces rapid dephosphorylation of Kv2.1, increasing its lateral translocation and dispersing the channel, thus enhancing its activation and controlling the homeostatic plasticity of the neuron (Misonou et al., 2004). Moreover, synaptic plasticity reduces I_h_ currents in the dendrites, normalizing membrane resistance along the dendrites (Campanac et al., 2008). This mechanism of regulating excitability is widely distributed across several neuronal types, brain areas, and channels (Campanac et al., 2008; Grubb and Burrone, 2010; Lezmy et al., 2017; Puhl et al., 2023). Conversely, TRPM4 trafficking has been widely described in non-excitable cells (Crnich et al., 2010; Cho et al., 2014; Rixecker et al., 2016; Bianchi et al., 2018; Blanco et al., 2019; Hwang et al., 2023), but there is no information about neurons and how stimuli such as cholinergic transmission modulate its traffic and function.

Here, we investigated whether cholinergic stimulation alters the distribution of TRPM4 in neurons using a combination of electrophysiological recordings, Ca^2+^ imaging, immunofluorescence, and fluorescence recovery after photobleaching (FRAP) analysis. We found that the cholinergic-induced increase in firing frequency is sensitive to CBA and 9-Ph, two TRPM4 inhibitors. Moreover, TRPM4 inhibition reduces the carbachol-induced increase in intracellular calcium. The increase in excitability is related to a reduction in the retrograde trafficking of TRPM4 and a decrease in its mobility in the plasma membrane. Together, these results indicate that cholinergic stimulation reorganizes TRPM4 in the plasma membrane to modulate its excitability, likely by increasing TRPM4 availability in the plasma membrane.

## Methods

All experiments were conducted according to animal protocols approved by the Ethics Committee of the Universidad de Santiago de Chile (N° 301/2018), following the rules and guidelines of the National Research and Development Agency (ANID). Male and female C57BL/6J mice were housed in a temperature- and humidity-controlled facility with a 12/12 h light/dark cycle, with water and food *ad libitum*.

### Cortical neuron culture

Primary frontal cortical neurons were prepared from E18 C57BL/6J mouse embryos. The frontal cortices were dissected in Hank’s balanced salt solution (HBSS) and digested in trypsin (0.25%) plus DNase I (0.03 mg/mL) for 8 min at 37°C, triturated, and plated in Minimum essential media (MEM) supplemented with horse serum (10%), glucose (0.1%), sodium pyruvate (0.5 mM), HEPES (10 mM), and penicillin-streptomycin (100 I.U./mL). Neurons were plated at a density of 50,000 cells per well on 12 mm coverslips pre-coated with poly-D-lysine (30 μg/mL) and laminin (2 μg/mL). After 6 h of plating, the media was replaced with Neurobasal medium supplemented with B27 (2%), GlutaMAX-I (1%), and penicillin-streptomycin (100 I.U/mL). Neurons were cultured at 37°C with 5% CO_2_. Half of the culture media was replaced every 3 days. Experiments were performed between days *in vitro* (DIV) 14–18.

### Electrophysiology

Neurons were recorded in Krebs buffer (in mM): 140 NaCl, 5 KCl, 1.3 MgCl, 2.5 CaCl_2_, 10 HEPES, 11 Glucose, pH 7.4, and ∼300 mOsm/kg. Neurons were placed in a recording chamber and mounted on a Nikon Ti2 microscope. They were continuously perfused with Krebs (2–3 mL/min) at 34°C ± 2°C. Whole-cell recordings were performed from cortical pyramidal neurons between DIV14-18 using borosilicate glass pipettes pulled to a resistance between 4–6 MΩ. For current-clamp recordings, the intracellular solution contained (in mM): 130 potassium-gluconate, 10 KCl, 10 HEPES, 0.5 EGTA, 2 Mg-ATP, 0.3 Na-GTP, 10 phosphocreatine, with pH 7.2 adjusted with KOH (∼300 mOsm/kg). The liquid junction potential was 16.4 mV, calculated using LJPcalc (https://swharden.com/LJPcalc) and not subtracted from the recordings. Cells showing changes >20% in the series resistance (R_s_) were discarded from the analysis. Current-clamp recordings were performed using an Axopatch 200B and digitized using a National Instruments PCIe-6323. Data was filtered at 10 kHz and acquired at 50 kHz using WinWCP5.7 (https://github.com/johndempster/WinWCPXE/releases/tag/V5.7.8). TRPM4 was inhibited using 10 µM 4-Chloro-2-[[2-(2-chlorophenoxy)acetyl]amino]benzoic acid (CBA), and 10 µM 9-Phenanthrol (9-Ph).

### Ca^2+^ imaging

Neurons were incubated with 3 µM Fluo4-AM in the Krebs buffer for 30 min at RT. Then, excess Fluo4-AM was washed out by incubating the neurons with Krebs buffer for 20 min at RT before imaging. Images were acquired using a Nikon TE300 attached to a Lambda DG-4 and controlled by Micromanager 2.0 through an Arduino interface (Edelstein et al., 2014). Changes in [Ca^2+^]_i_ were observed using a ×40 objective (PlanNeofluar, 0.75N.A.) during exposure to 470 nm, and the intensity of the fluorescence emission at 505 nm was recorded using an Orca-ER CCD camera. Neurons were constantly perfused at 2–3 mL/min with Krebs buffer at 34°C ± 2°C. The fluorescence intensity was calculated using Fiji-ImageJ, and the results are presented as the ratio of normalized fluorescence [(F-F_0_)/F_0_].

### Immunofluorescence and membrane staining

Neurons were fixed in 4% paraformaldehyde for 15 min at room temperature and then washed in phosphate buffered saline (PBS). Subsequently, the cells were permeabilized for 5 min with 0.1% Triton X-100 and blocked for 1 h in 10% goat serum. Neurons were then incubated with the primary antibodies diluted in 10% goat serum: EEA1 (Early endosome A1 antigen, 1:500; Synaptic Systems RRID:AB_2744647) and TRPM4 (1:100; RRID:AB_2040250) overnight at 4°C. Afterward, they were incubated with the appropriate secondary antibodies: Goat anti-Guinea Pig IgG, Alexa Fluor 555 (1:1,000; ThermoFisher RRID:AB_2535856), and Donkey anti-rabbit IgG, Alexa Fluor 488 (1:1,000; ThermoFisher RRID:AB_2535792) for 1 h at room temperature and washed. Coverslips were mounted in Prolong Gold. For membrane staining, after immunolabeling with the TRPM4 antibody, coverslips were incubated with the membrane dye CellBrite Orange (1:200, Biotium) for 15 min at RT and washed three times in PBS. The coverslips were then imaged in PBS. Images were acquired using a laser scanning confocal microscope (Zeiss LSM 800) with proper excitation and emission filters, pinhole set to 1, and a 40x (1.4NA) oil immersion objective. Laser power and gain settings were adjusted to prevent signal saturation.

### Cortical neuron transfections

Neurons were transfected using Lipofectamine 2000 (Invitrogen/Thermo Fisher) between DIV 5–7 with the pEGFP-N1-mTRPM4 vector encoding mouse TRPM4 fused with EGFP protein at the N-terminal under the control of the cytomegalovirus promoter (TRPM4-EGFPN1) (Gerzanich et al., 2009). Briefly, 1 µg DNA was mixed with 2.5 µL Lipofectamine (ratio 1:25) in 50 µL non-supplemented Neurobasal (ratio 1:50) and incubated for 20 min at room temperature. Neuron media were saved and replaced with fresh non-supplemented neurobasal. The mixture was then added to the neurons and incubated for 1 h at 37°C with 5% CO_2_. Next, the transfection media was removed, neurons were washed with fresh neurobasal, the original media was added, and neurons were maintained at 37°C in 5% CO_2_ until the experiments.

### Fluorescence recovery after photobleaching (FRAP)

EGFP-TRPM4 recovery after photobleaching (FRAP) experiments were performed using a Zeiss LSM 800 confocal microscope with a ×63 objective with a numerical aperture of 1.4 and digital zoom of 3. One region of interest (ROI) on the plasma membrane was selected and bleached for 10 s with a 488 nm laser at 1 W. The fluorescence was measured for 3 min with image acquisition set at 2 s. Cch stimulation was performed 2 s after the photobleaching and kept until the end of the image acquisition. Neurons were constantly perfused at 2–3 mL/min with a Krebs buffer at 34°C ± 2°C using a gravity-driven perfusion system.

### FRAP analysis

The mean ROI intensity of the bleached area was obtained and corrected with background values and the bleaching during image acquisition. Data were normalized with control fluorescence averaged over 10 initial frames before bleaching and stated as one intensity. Correction and normalization were performed using the easyFRAP script, using Matlab (Rapsomaniki et al., 2012). Recovery time was calculated by an exponential one-phase association fit (increasing exponential). The mobile phase value was calculated as plateau/maximum fluorescence.

### Colocalization image analysis

Colocalization analysis was conducted in Fiji ImageJ using the EzColocalization plugin (Stauffer et al., 2018). Using the plugin, we defined a somatic ROI (for early endosome) or a ROI surrounding the membrane (for plasma membrane) in each image of the stack, and we obtained the Pearson’s correlation coefficient (PCC) and Manders’ colocalization coefficients 1 and 2 (M1 and M2), setting the threshold using the Costes algorithm.

### Data analysis

All numbers of experiments are indicated in the figure caption as the number of individual neurons (n) and the neuron cultures (N); in some graphs it is shown the number of individual cultures as solid color symbols and in open symbols and light color the individual cells from each culture. Electrophysiological data were analyzed using Python 3.7. Data are reported in the text as mean ± standard deviation and in the graph as a median±95% confidence interval (C.I.), unless stated otherwise. Statistical significance between group means was evaluated using one-way ANOVA followed by multiple comparison *post hoc* tests, in some cases, *t*-tests were used. Statistical significance was determined using a *p* < 0.05. All statistical tests were performed on GraphPad Prism 7.

## Results

### TRPM4-dependent increase in firing frequency after cholinergic stimulation

**FIGURE 1:**
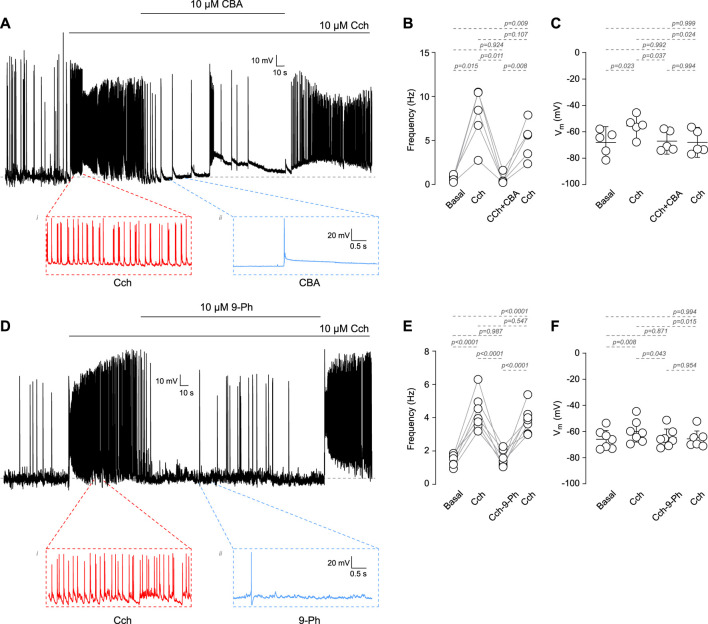
Cholinergic dependent increase in excitability mediated by TRPM4. **(A)** Representative voltage recordings at cortical pyramidal neurons at DIV14-18, top trace shows the AP evoked by 10 µM Cch and the effect of 10 µM CBA, bottom traces show a zoomed area of the events recorded. **(B)** Summary graph of the frequency of the AP and the RMP **(C)** at basal (n = 5, N = 5), 10 µM Cch (n = 5, N = 5), 10 µM Cch+10 µM CBA (n = 5, N = 5) and the recovery after CBA (n = 5, N = 5). **(D)** Representative voltage trace showing the effect of 10 µM Cch and 10 µM Cch+10 µM 9-Ph, bottom traces show a zoomed area of the events recorded. **(E)** Summary graph of the frequency of the AP and the RMP **(F)** at basal (n = 7, N = 7), 10 µM Cch (n = 7, N = 7), 10 µM Cch+10 µM 9-Ph (n = 7, N = 7), and the recovery after 9-Ph (n = 7, N = 7). In **(C,F)** data are presented as the mean ±95 C.I. *p-*values are shown above each graph. Statistical significance was evaluated using one-way ANOVA, with a Tukey *post hoc*.

Among the TRP family, only TRPM4 and TRPM5 are directly activated by Ca^2+^_i_ (Ullrich et al., 2005). These channels play critical roles in the afterdepolarization potential, firing frequency, and the resting membrane potential (RMP). However, it is not clear whether TRPM4 participates in cholinergic-induced increases in AP firing. To address this question, we recorded the activity of cortical pyramidal neurons in DIV14-18 in response to the cholinergic agonist carbachol (Cch) and in the presence of the TRPM4 pharmacological inhibitors CBA or 9-Ph (Figure 1). We observed that 10 µM Cch increases the firing frequency approximately tenfold; this effect is reduced by the perfusion of 10 µM CBA (Basal = 0.58 ± 0.34 Hz, Cch = 6.04 ± 4.02 Hz, Cch + CBA = 0.75 ± 0.89 Hz, Figures 1A, B). Moreover, we observed that Cch depolarizes the RMP by approximately 13 mV, but the application of 10 µM CBA restores the RMP to its basal levels (Basal = −68.1 ± 9.7 mV, Cch = −55.5 ± 8.03 mV, Cch + CBA = −67.1 ± 7.9 mV, Figure 1C) but is not recovered to the basal levels after CBA washout. Additionally, we tested 9-Ph; we found that this inhibitor produces a similar effect as CBA on both, the frequency (Basal = 1.6 ± 0.14 Hz, Cch = 4.8 ± 1.1 Hz, Cch+9-Ph = 1.7 ± 0.55 Hz, Figures 1D, E) and RMP (Basal = −69.9 ± 7.4 mV, Cch = −59.9 ± 8.7 mV, Cch+9-Ph = −64.7 ± 7.2 mV, Figure 1F), moreover, we found that Cch effect in frequency was recovered after drugs washout but not the RMP. Additionally, continuous Cch stimulation shows no statistically significant rundown of the activity (Supplementary Figure S1A), supporting the observed effect due to TRPM4 inhibition. To determine the involvement of synaptic activity in the effect of Cch, we measure the spontaneous activity in the presence of 0.5 µM TTx and 10 µM Cch and measure the AP firing, we found that TTx completely reduce basal activity and obliterates the effect of Cch in firing (Basal = 1 ± 1.1 Hz, TTx = 0.1 ± 0.2 Hz, TTx + Cch = 0.001 ± 0.002 Hz, Supplementary Figure S1B), Together, these data indicates that TRPM4 contributes to the cholinergic-induce increase in AP firing and suggest a combination between synaptic activity and intrinsic excitability in the origin of the AP firing.

### TRPM4 inhibition reduces cholinergic-induced intracellular Ca^2+^ increase

**FIGURE 2:**
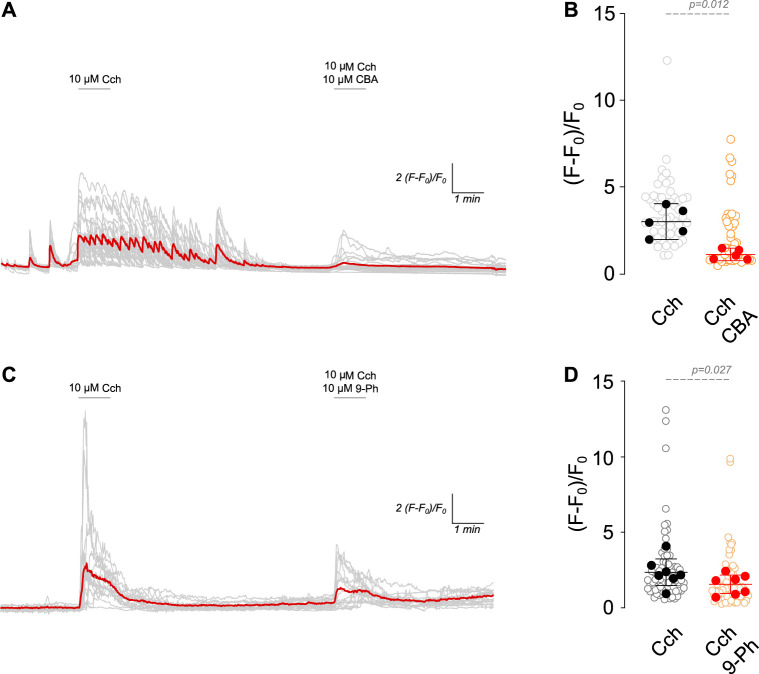
TRPM4 inhibition reduces cholinergic induced Ca^2+^_i_ increase. **(A)** Representative Ca^2+^ imaging trace showing the increase in the fluorescence after 10 µM Cch stimulation for 1 min following a washout and the coapplication of 10 µM Cch+10 µM CBA for 1 min. **(B)** Summary graph showing the quantification of the peak of the Ca^2+^ signal (n = 56, N = 5). **(C)** Representative Ca^2+^ imaging trace showing the increase in the fluorescence after 10 µM Cch stimulation for 1 min following a washout and the coapplication of 10 µM Cch+10 µM 9-Ph for 1 min. **(D)** Summary graph showing the quantification of the ratio of fluorescence of the peak of the Ca^2+^ signal (n = 77, N = 7). Red traces showed the averaged signal, gray traces show the individual cells recorded in all experiments. In **(B,D)** data are presented as the mean ±95 C.I. Statistical significance was tested using paired *t*-test.

TRPM4 is activated by Ca^2+^_i_ but is impermeable to Ca^2+^. This characteristic enables it to control intracellular calcium levels by fluctuating the Ca^2+^ driving force in non-excitable cells (Launay et al., 2004) and through the activation of voltage-gated calcium channels (VGCCs) by its effect on the RMP in excitable cells (Fliegert et al., 2007). In this regard, cholinergic stimulation increases Ca^2+^_i_ through nicotinic receptors or G_q_ protein-coupled muscarinic receptors (via IP_3_R) (Colangelo et al., 2019). Since TRPM4 participates in the increase in firing rate after cholinergic stimulation, we hypothesized that TRPM4 inhibition must decrease intracellular calcium levels. To corroborate this hypothesis, we measured intracellular calcium levels in response to 10 µM Cch (1 min) and then in the presence of 10 µM Cch with 10 µM CBA or 10 µM 9-Ph (Figure 2). We found that Cch induces a robust response, showing Ca^2+^_i_ oscillations that last several minutes after the Cch washout. The perfusion of Cch + CBA reduces Cch-induced maximal response by 2.6 times (Cch = 3.01 ± 0.83, Cch + CBA = 1.12 ± 0.3, Figures 2A, B). Similarly, we found that 9-Ph reduces the Cch-induced increase in Ca^2+^_i_ by 1.5 times (Cch = 2.32 ± 0.9, Cch+9-Ph = 1.5 ± 0.67, Figures 2C, D), these changes are not related to a change induced by repetitive stimulation as two pulses of 10 µM Cch produce similar maximal responses (Supplementary Figure S1C). Together, these results indicate that TRPM4 activation regulates cholinergic-induced Ca^2+^_i_ increase.

### Cholinergic stimulation reduces TRPM4 recycling

**FIGURE 3:**
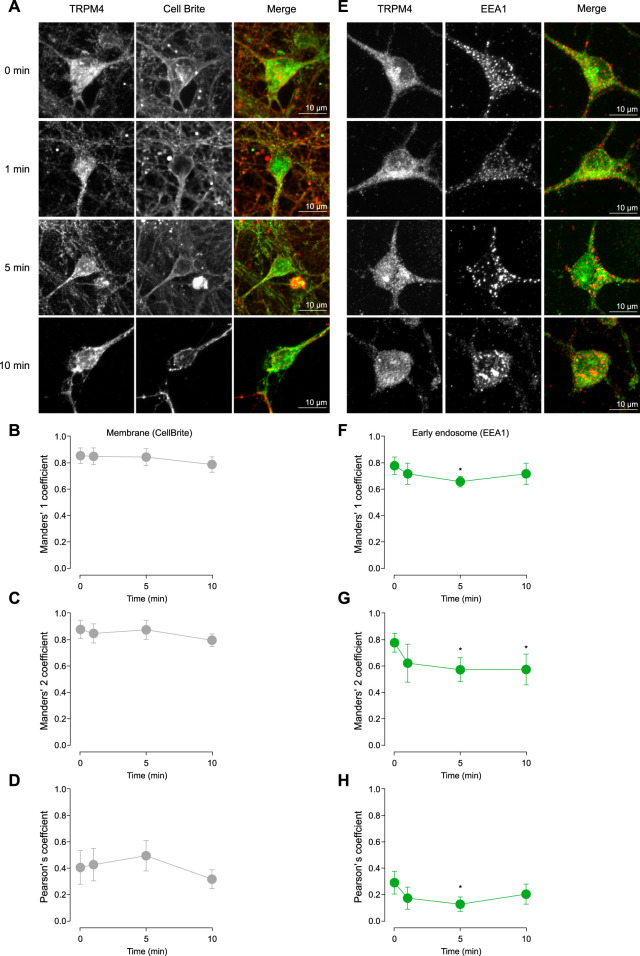
TRPM4 trafficking after cholinergic stimulation. **(A)** Representative confocal image of pyramidal neurons in culture at DIV14 showing the effect of 10 µM Cch at 0, 1, 5, and 10 min in the TRPM4 localization in the plasma membrane using the Cell Brite membrane marker. **(B)** Shows the Manders’ 1, **(C)** Manders’ 2 and **(D)** Pearson’s coefficient for TRPM4-CellBrite colocalization. **(E)** Representative confocal image showing the effect of 10 µM Cch at 0, 1, 5, and 10 min in the TRPM4 localization in the early endosome using the EEA1 marker. **(F)** Shows the Manders’ 1, **(G)** Manders’ 2 and **(H)** Pearson’s coefficient for TRPM4-EEA1 colocalization. Data is presented as the mean ± 95% C.I, stars indicate statistical significance between 0 min and the time indicated, statistical significance was tested using one-way ANOVA with a Dunnett’s *post hoc* test, *n* = 22, *N* = 5. “*” in the graphs indicates *p* < 0.05.

The Cch-induced effects on TRPM4 in the firing frequency observed in cortical neurons could be explained by changes in the trafficking of the channel. To address this hypothesis, we treated cortical neurons with saline or 10 µM Cch for 1, 5, or 10 min. Then, we fixed and immunostained the neurons for TRPM4 along with either Cell Brite (plasma membrane marker) or EEA1 (early endosome marker). After this, we quantified the colocalization using Pearson’s, Manders’ 1 (M1), and Manders’ 2 (M2) correlation coefficients. We used M1 coefficients to assess the overlap between the plasma membrane and TRPM4, and M2 for the fraction of TRPM4 that overlaps with the plasma membrane. For both coefficients we obtained values higher than 0.5, indicating a medium to high colocalization rate, the Cch stimulation has no effect in the colocalization (M1, 0 min = 0.85 ± 0.12, 1 min-Cch = 0.85 ± 0.1, 5 min-Cch = 0.84 ± 0.13, 10 min-Cch = 0.79 ± 0.11; M2, 0 min = 0.88 ± 0.14, 1 min-Cch = 0.89 ± 0.12, 5 min-Cch = 0.875 ± 0.15, 10 min-Cch = 0.795 ± 0.08, Figures 3A–C). Moreover, we also used the Pearson correlation coefficient and found values around 0.4, indicating medium to low correlation of the signals and Cch had no effect in this parameter (0 min = 0.41 ± 0.25, 1 min-Cch = 0.43 ± 0.2, 5 min-Cch = 0.49 ± 0.23, 10 min-Cch = 0.32 ± 0.13, Figures 3A, D).

Next, we measured the colocalization of TRPM4 and EEA1. With M1, we evaluates the amount of EEA1 that is present in the TRPM4 signal, we found a decrease at 5 min Cch, indicating that EEA1 colocalization with TRPM4 is reduced, suggesting a decrease in the amount of EEA1 containing vesicles or an increase of TRPM4 in other areas (0 min = 0.78 ± 0.15, 1 min Cch = 0.72 ± 0.18, 5 min-Cch = 0.66 ± 0.09, 10 min-Cch = 0.72 ± 0.19, Figures 3E, F). With M2, we found a decrease in the colocalization at 5 and 10 min post Cch (0 min = 0.77 ± 0.16, 1 min-Cch = 0.62 ± 0.29, 5 min-Cch = 0.57 ± 0.21, 10 min-Cch = 0.57 ± 0.25, Figures 3E, G), this data indicates that TRPM4 is less colocalized with EEA1, suggesting that TRPM4 decreases its endocytosis after Cch stimulation. Moreover, we also used the Pearson correlation coefficient and found a lower correlation between TRPM4 and EEA1 (0 min = 0.29 ± 0.2, 1 min-Cch = 0.17 ± 0.17, 5 min-Cch = 0.13 ± 0.13, 10 min-Cch = 0.2 ± 0.18, Figures 3E, H), which is expected as the majority of the TRPM4 signal is outside of the vesicles containing EEA1. Together, these results indicate that Cch stimulation reduces TRPM4 recycling while sustaining a constant level of TRPM4 in the plasma membrane, suggesting that cholinergic stimulation affects the stability of TRPM4 in the plasma membrane.

### Cholinergic stimulation reduces TRPM4 membrane mobilization

**FIGURE 4:**
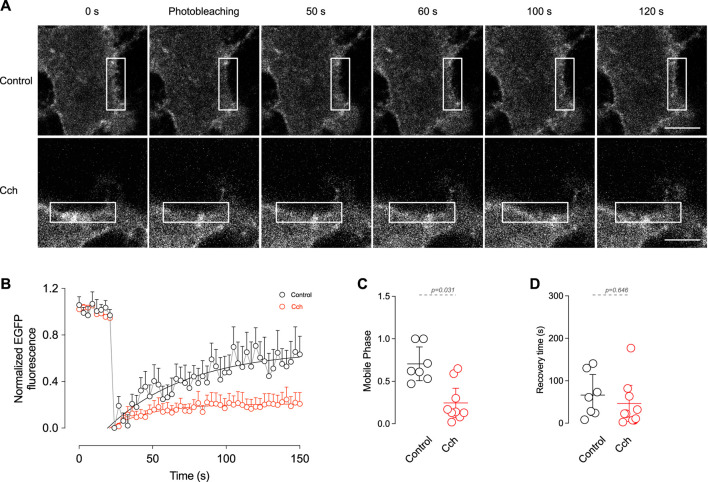
*In vitro* FRAP shows the mobile fraction of TRPM4 in the plasma membrane. **(A)** Representative images of pyramidal neurons expressing EGFP-N1-TRPM4 and the time course of fluorescence recovery in control and Cch stimulated condition. **(B)** Summary graph showing the time course of the fluorescence in control (N = 7) and in response to 10 µM Cch (N = 9). Solid lines represent the single exponential fitting. **(C)** Shows the summary graph of the mobile phase and **(D)** the recovery time constant. In **(B)** data is presented as the mean ± SD and in **(C,D)** is presented as the mean ±95% C.I. *p*-values are shown above the graph and the statistical significance was tested using unpaired *t*-test.

The observation that cholinergic stimulation has no effect on TRPM4 content in the plasma membrane but reduces its recycling in the early endosome indicates that cholinergic stimulation has a low effect on TRPM4 trafficking. Despite this, it does not rule out the possibility of dynamic changes of TRPM4 within the membrane, such as lateral diffusion. To determine the effect of cholinergic stimulation on TRPM4 kinetics in the plasma membrane, we performed FRAP experiments in cortical pyramidal neurons expressing EGFP-TRPM4 at DIV14. Following the bleaching (10 s), we found that non-stimulated neurons present a highly mobile TRPM4 fraction with a time of recovery that fits to a single exponential. The application of Cch reduces the mobile phase (Basal = 0.71 ± 0.21, Cch = 0.28 ± 0.25, Figures 4A–C) with no changes in the recovery time constant (Basal = 66.5 ± 52.02 s, Cch = 53.9 ± 61.7 s, Figures 4A, B, D). This data indicates that a significant portion of TRPM4 is engaged in an immobile fraction and after cholinergic stimulation this fraction increases, suggesting that they are not readily interchanged, likely as a result of a diffusion restriction via protein interaction and/or molecular crowding within the plasma membrane of the neuron.

## Discussion

Here, we demonstrate that cholinergic stimulation increases pyramidal neuron firing through a mechanism dependent on TRPM4, furthermore, TRPM4 inhibition reduces cholinergic induced Ca^2+^_i_ increase. Interestingly, the increase in firing is accompanied by a reduction in TRPM4 recycling and an immobilization of the channel in the plasma membrane. These observations indicate that cholinergic transmission stabilizes TRPM4 in the plasma membrane, enhancing its availability during neuronal activity, suggesting a role in the modulation of neuronal response.

The recording of neuronal firing after cholinergic stimulation revealed a steady increase in action potential (AP) firing frequency. However, TRPM4 inhibition reduces AP firing, suggesting that TRPM4 participates in the sustained AP firing. Several studies support this, proving that TRPM4 plays a role in AP firing, like in preBötzinger neurons, where it mediates I_CAN_, and its inhibition reduces the burst firing magnitude during respiratory rhythm (Picardo et al., 2019). Similarly, O'Malley et al. (2020) showed that TRPM4 drives the I_CAN_ responsible for the plateau potential driving the persistent firing in the thalamic reticular nucleus neurons.

The aforementioned observations are generated by cell-intrinsic firing mechanism, however, TRPM4 also participates in the synaptic driven firing, in this regard, TRPM4 inhibition reduces firing induced by mGluR type 1 activation in preBötzinger neurons (Mironov, 2008), contributes to the afterdepolarization induced by high-frequency synaptic stimulation, increasing AP firing frequency in pyramidal neurons in layer 2/3 of the medial prefrontal cortex (Riquelme et al., 2021) and partially contributing to the muscarinic-induced slow afterdepolarization in layer five pyramidal neurons of the prefrontal cortex, suggesting that TRPM4 participates in regulating AP firing in response to increased intracellular Ca^2+^ (Lei et al., 2014).

In our experiments, the effect of cholinergic stimulation on AP firing could be direct, through the activation of cholinergic receptors in postsynaptic neurons, or indirect, through the activation of cholinergic receptors in presynaptic neurons leading to increased glutamate release onto postsynaptic neurons. Our results suggest a mixed effect, as cholinergic activation in the presence of TTx did not trigger APs but induced a small depolarization. In this context, synaptic activity may start AP firing, while TRPM4 may sustain it. However, further investigation is needed to fully determine the underlying mechanism of this effect.

While we cannot rule out off-target effects of the inhibitors, to our knowledge, only one study describes off-target effects of 9-Ph in TMEM16A calcium-activated chloride channels. However, we did not observe a depolarization with 9-Ph as expected by a Cl^−^ channel inhibition in neurons (Burris et al., 2015). On the other hand, a recent study indicates that CBA has a species-specific effect on TRPM4, inhibiting human TRPM4 but having no effect on mouse TRPM4 overexpressed in TsA201 kidney cells (Prakash et al., 2021). However, the experimental conditions differed from those used in our experiments and others reported previously. The characterization was performed in the inside-out patch clamp, where several intracellular components are missing. Additionally, the experiments were performed at room temperature. This is a key point because a recent study indicates that TRPM4 is inhibited by ATP at 20°C–22°C but is less sensitive at 37°C (Hu et al., 2024). Contrary to this, in our experiments presented in this work and in two other studies (O’Malley et al., 2020; Combe et al., 2023), CBA shows inhibitory effects on TRPM4 in mouse neurons in experiments performed in whole-cell and at 37°C. In this context, our results strongly suggest that TRPM4 participates in the initiation of AP firing following cholinergic stimulation.

TRPM4 has been widely described as a modulator of the Ca^2+^_i_ during periods of activity in different cell types. While most studies indicates that the absence or inhibition of TRPM4 increases Ca^2+^_i_ amplitude, while obliterating Ca^2+^_i_ oscillations (Launay et al., 2002; 2004; Cheng et al., 2007; Shimizu et al., 2009) its effect is highly dependent on the RMP, i.e., in non-excitable cells, TRPM4 inhibition increases Ca^2+^_i_ (Fliegert et al., 2007), but in neurons, it reduces Ca^2+^_i_ through the activation of VGCC channels (Li et al., 2021).

Moreover, since our cortical culture are a mix of different types of neurons, we observed heterogeneous responses, like oscillatory behavior in ∼34% of the cells, in this regard, the effect of TRPM4 inhibition appears to be both, a reduction in the peak response and a disruption of the Ca^2+^_i_ oscillation, both responses are in line with the reported effect of TRPM4 inhibition in non-neuronal cells.

In isolated atrial cardiomyocytes, the inhibition or knockdown of TRPM4 reduces AP duration by 50% (Simard et al., 2013), and reduces the AP frequency in sinoatrial node cells (Demion et al., 2007; Hof et al., 2015), and mutation impairing endocytosis produce progressive familial heart block type 1 (Kruse et al., 2009). Moreover, in artery smooth muscle cells, the activation of TRPM4 increases the contraction of the artery and is regulated by IP_3_R which increases Ca^2+^ in nanodomains formed by the sarcoplasmic reticulum and plasma membrane junctions. While this effect has not been described in neurons, the activation of TRPM4 through receptors inducing IP_3_R has been extensively described (Launay et al., 2002; Gonzales and Earley, 2012; Gonzales et al., 2014; Provence et al., 2017). On the other hand, TRPM4 participates in the smooth muscle membrane depolarization inducing the activation of VGCC and inducing contraction, this effect is mediated by an increase in trafficking induced by the activation of PKCδ (Crnich et al., 2010). Moreover, in pancreatic beta cells, glucose induces the activation of TRPM4 depolarizing the cell further than the K_ATP_ closure and activating VGCC thus producing pulsatile insulin secretion (Cheng et al., 2007), in neurons, the activation of TRPM4 depolarizes neurons and participates in the activation of VGCC. These effects in the excitability of non-neuronal cells are like those observed in neuronal cells, and as such the traffic of TRPM4 channel plays a critical role in its physiological function.

Changes in TRPM4 localization and distribution are critical for its activity and impact neuronal physiology. In this regard, TRPM4 trafficking has been extensively investigated in non-excitable cells where it participates in epithelial cell migration and wound healing (Blanco et al., 2019), and insulin vesicle release (Cheng et al., 2007), however its role in neuronal physiology is not completely understood. Our experiments indicate that cholinergic stimulation reduces TRPM4 recycling, while the plasma membrane content remains constant, suggesting that TRPM4 in non-stimulated conditions is constantly recycled but when engaged in cholinergic neuronal activity, it remains stable in the plasma membrane, favoring neuronal activity.

Similarly, synaptic activity stabilizes AMPA receptor in the synaptic terminal and induces receptor lateral movement which populate the synapsis after long term potentiation (Heine et al., 2008), moreover, this receptor has a tunable highly mobile and immobile fraction that allow neurons to respond to different stimulus (Chen et al., 2021). In the case of Kv2.1, neuronal activity induces a declustering of the channel and a repopulation of the membrane, keeping the channel away from signaling complexes and avoiding its phosphorylation, thus increasing the excitability of the neurons (Misonou et al., 2004). In this regard, TRPM4 activation in CA1 neurons requires muscarinic activation and depolarization-induced Ca^2+^_i_ increase, with no involvement of IP_3_R or RyR, to induce an acceleration of the AP firing (Combe et al., 2023). Similarly, our results suggest that TRPM4 traffic is highly regulated by cholinergic stimulation.

The localization of the channels is critical for its neuronal physiology, through local regulation of the membrane potential, excitability and by placing the channels in the proximity of local signaling complexes that can modulate its activity or expression. Our data indicates that cholinergic stimulation stabilizes TRPM4 in the plasma membrane, reducing its mobility. This data suggests that cholinergic stimulation may engage TRPM4 in signaling pathways or increase its association with cytoskeleton proteins that can facilitate TRPM4 activity. Moreover, our results suggest that TRPM4 may be forming microdomains after cholinergic stimulation. The observation that TRPM4 is locked in the membrane came from the increase in the immobile phase in the FRAP experiments; previous reports using TIRF-FRAP indicates that TRPM4 in non-stimulated condition recovers its signals mainly by the incorporation of vesicles carrying TRPM4 but no through lateral diffusion (Ghosh et al., 2014). Our results showed no increase in TRPM4 signal in the plasma membrane related to the control, but a reduction in early endosome, strongly suggesting that Cch does not increase vesicle fusion but reduces mobility and recycling.

This study indicates that TRPM4 is activated by cholinergic transmission and induce an increase in the channel locked in the plasma membrane and reducing the channel recycling, this produces an efficient increase in excitability and its inhibition affect the global excitability of the neuron, reducing the cholinergic-induced increase in Ca^2+^_i_, thus reducing excitability at all levels. These results implicate that TRPM4 is critical for oscillatory behavior in several brain regions, thus giving clues to a new level of control of the excitability in a Ca^2+^ dependent way modulated by acetylcholine and potentially other neuromodulators, providing an added level of control during neuronal activity.

## Data Availability

The raw data supporting the conclusions of this article will be made available by the authors, without undue reservation.
